# Score for the Overall Survival Probability of Patients With Pancreatic Adenocarcinoma of the Body and Tail After Surgery: A Novel Nomogram-Based Risk Assessment

**DOI:** 10.3389/fonc.2020.00590

**Published:** 2020-04-28

**Authors:** Chaobin He, Shuxin Sun, Yu Zhang, Xiaojun Lin, Shengping Li

**Affiliations:** ^1^State Key Laboratory of Oncology in South China, Department of Pancreatobiliary Surgery, Collaborative Innovation Center for Cancer Medicine, Sun Yat-sen University Cancer Center, Guangzhou, China; ^2^State Key Laboratory of Ophthalmology, Retina Division, Zhongshan Ophthalmic Center, Sun Yet-sen University, Guangzhou, China

**Keywords:** pancreatic adenocarcinoma, nomogram, overall survival, prognosis, SEER

## Abstract

Pancreatic adenocarcinoma of the body and tail often has a dismal prognosis and lacks a specific prognostic stage. The aim of this study was to construct a nomogram for predicting survival of patients with pancreatic adenocarcinoma of the body and tail after surgery. Data of patients were selected from the Surveillance, Epidemiology, and End Results (SEER) database and from medical records of Sun Yat-sen University Cancer Center (SYSUCC). In a multivariate analysis for overall survival (OS), the following six variables were identified as independent predictors and incorporated into the nomogram: age, tumor differentiation, tumor size, lymph node ratio (LNR), and chemotherapy. A nomogram was built based on independent risk predictors. The concordance index (C-index) for nomogram, Tumor-Node-Metastasis (TNM) 7th and 8th stage system were 0.775 [95% confidence interval (CI), 0.731–0.819], 0.617 (95%CI, 0.575–0.659), and 0.632 (95%CI, 0.588–0.676), respectively. The calibrated nomogram predicted survival rates which closely corresponded to the actual survival rates. Furthermore, the values of the area under receiver operating characteristic (ROC) curves (AUC) of the nomograms were higher than those of the TNM 7th or 8th stage system in predicting 1-, 2-, and 3-year survival of patients in training and external validation cohorts. The well-calibrated nomogram could be used to predict prognosis for patients with pancreatic adenocarcinoma of the body and tail after surgery.

## Introduction

Pancreatic ductal adenocarcinoma (PDAC), which represents one of the most common gastrointestinal tumors, is the fourth cause of cancer deaths in developed countries (1). Surgery leads to the best chance of survival with a 5-year overall survival (OS) rate of only 5–7% (2). Compared with PDAC of the head of the pancreas, the discrepancies of ontogeny would lead to significant differences in cell composition, blood supply, lymphatic and venous backflow in PDAC of the body and tail of the pancreas (3). Moreover, due to the lack of obstructive jaundice, PDAC which occurred at the body and tail has a lower resectable rate and a more dismal prognosis than PDAC of the head (4, 5).

Several independent prognostic factors of PDAC have been identified, such as lymph node (LN) metastasis, tumor size, and resection margin (6, 7). In these studies, the prognostic influence of risk factors on PDAC of the body and tail is only estimated because the reported predictors of prognosis mainly focus on PDAC occurring at the pancreatic head. It is known that the differences in ontogeny lead to significant differences in clinical characteristics between tumors occurred at the head or the body/tail (8). However, the 8th edition of the tumor-node-metastasis (TNM) staging system of the American Joint Commission on Cancer (AJCC), (9) which is commonly used to stage diseases, takes only common predictive factors into account and does not incorporate the tumor site. Furthermore, the 8th edition of the TNM is still cumbersome and not specifically designed to predict prognosis. The TNM staging system algorithm, which uses risk factors to make clinical decisions, needs to be validated. The lack of an accurate and reliable staging system for PDAC of the body and tail makes it difficult and challenging for doctors to appropriately identify patients at risk of long-term survival. Therefore, it is necessary to develop a technically feasible and an easily accessible clinical staging system to stratify the prognosis of patients with PDAC of the body and tail when surgery is urgently required.

Nomograms, which have been adopted in various cancers (10–13) and have shown favorable results, compared with traditional TNM staging systems, (14) are simple graphical depictions of the predictive model and are used to provide the probabilities of outcomes for individual patient (15). However, few studies have reported specific nomograms for patients with PDAC of the body and tail after surgery. The purpose of this analysis was to develop a clinically useful nomogram which could be used to predict the prognosis of patients with PDAC of the body and tail after surgery.

## Materials and Methods

### Study Design

In this retrospective study, according to the inclusion and exclusion criteria, suitable patients were selected and clinicopathological variables of these patients were retrospectively reviewed. Extensive evaluation of concerning risk factors were conducted using a univariate analysis for all included variables and a multivariate analysis was adopted to select the independent risk factors. A nomogram was built based on these independent risk factors and validated in both training and external validation cohorts. The predictive power of the established nomogram was also compared with that of the 7th and 8th editions of TNM stage systems. This study was approved by the Institutional Review Board (IRB) of the Sun Yat-sen University Cancer Center. Each individual participant from the SYSUCC database provided informed written consent. All procedures performed in studies involving human participants were in accordance with the 1964 Helsinki Declaration and its later amendments or comparable ethical standards.

### Patients

The Surveillance, Epidemiology, and End Results (SEER) program maintains the largest clinical dataset in the United States and provides data on cancer incidence and survival. For this research, the training cohort of patients with PDAC of the body and tail were obtained from the SEER database (2004–2015). The International Classification of Diseases for Oncology, Third Edition (ICD-O-3), histology code 8140/3 and site codes C25.1 and C25.2, were followed in the SEER database using SEER^*^Stat software version 8.3.4. The second cohort of patients was obtained from the SYSUCC (2009–2017), which was used as an external validation cohort. The inclusion criteria were as follows: (1) pathologically confirmed pancreatic adenocarcinoma and radiologically confirmed PDAC of the body and tail; (2) radical surgical resection. The following exclusion criteria were adopted: (1) second primary cancer; (2) distant metastases; (3) other treatments only, including chemotherapy or radiotherapy; and (4) missing or incomplete information. The information of TNM system was adopted in this study, which was in accordance with previous studies (16, 17).

### Data Collection

Pathological and clinical variables, such as age at diagnosis, gender, tumor size, tumor differentiation, TNM stage, chemotherapy, radiotherapy, and follow-up information, were extracted from the SEER and SYSUCC databases. Sixty was used as the cutoff value of age in this study. The time-dependent receiver operating characteristic (ROC) curve analysis was used to determine the optimal cutoff value for lymph node ratio (LNR), which was defined as the same as previous studies (18). OS was calculated from the date of surgery to the date of death or last follow-up.

### Statistical Analysis

The chi-square test and Fisher's exact test were used to compare categorical data, which are shown as frequencies and proportions. Variables that were significantly associated with OS were analyzed in the multivariate analysis using the Cox regression model to determine the independent predictive factors, along with the corresponding 95% confidence interval (CI). Survival differences were compared with log-rank test. The nomogram was developed according to previous protocols (18–20) and the area under the ROC curve (AUC) was used to evaluate and compare the precision of predicting 1-, 2-, and 3-year survival with the nomogram. All statistical analyses were performed using R version 3.4.2 software (The R Foundation for Statistical Computing, Vienna, Austria. http://www.r-project.org). A two-tailed *P*-value was considered statistically significant if < 0.05.

## Results

### Patient Characteristics

**Figure 1:**
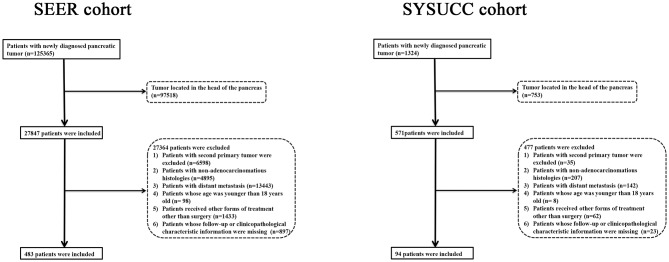
The flow diagram of the selection process for the study cohort.

The work flow of the current study is shown in Figure 1. The data of a total of 483 eligible patients with PDAC of the body and tail after surgery were obtained from the SEER database. There were 213 patients with PDAC of the body, and the other 270 patients had PDAC of the tail. A summary of baseline characteristics of the patients is shown in Table 1. The median age of patients in the SEER database was 65 years (range: 32–91 years). Of these patients, 238 (49.3%) were male. A tumor size larger than 4 cm (222; 46.0%) was the most common size. Moderately differentiated tumors (242; 50.1%) were most common, followed by poorly differentiated (194; 40.2%) and well-differentiated tumors (47; 9.7%). A total of 252 (52.2%) patients had LN metastasis. Most patients (168; 34.8%) were categorized as TNM stage III, 29.6% (143) were stage IIB, 12.0% (58) were stage IIA, and 23.6% (114) were stage I. In addition, the proportions of patients with LN metastasis and higher LNR values were higher in patients with PDAC of the tail. Other than these two variables, the other characteristics, including age, gender, race, tumor size, tumor differentiation, and TNM stage (8th edition), were comparable. LNR, with a cutoff value of 0.118, showed the greatest predictive power and was used to analyze survival in this study (Supplementary Figure 1). A total of 230 (47.6%) patients had received chemotherapy while 141 (29.2%) patients received radiotherapy in this study.

**Table 1:** Characteristics and overall survival of patients with pancreatic adenocarcinoma of the body and tail.

**Characteristics**		**SEER cohort**					**SYSUCC cohort**				
		**Patients**		**Tumor site**		***P***	**Patients**		**Tumor site**		***P***
				**Body**	**Tail**				**Body**	**Tail**	
Total		483	100.0	213	270		94	100.0	41	53	
Age	<60 years	318	65.8	137	181	0.563	72	76.6	29	43	0.326
	≥ 60 years	165	34.2	76	89		22	23.4	12	10	
Gender	Male	238	49.3	106	132	0.855	52	55.3	20	32	0.300
	Female	245	50.7	107	138		42	44.7	21	21	
Race	White	378	78.3	177	201	0.052					NA
	Black	57	11.8	17	40						
	Asian	48	9.9	20	28						
Tumor size	≤ 2 cm	76	15.7	37	39	0.107	13	13.8	3	10	0.274
	2 ~ 4 cm	185	38.3	90	95		28	29.8	13	15	
	> 4 cm	222	46.0	87	135		53	56.4	25	28	
Tumor differentiation	Well	47	9.7	18	29	0.510	6	6.4	3	3	0.948
	Moderately	242	50.1	98	144		81	86.2	35	46	
	Poorly	194	40.2	89	105		7	7.4	3	4	
LN metastasis (7th edition)	Absent	231	47.8	120	111	0.002	86	91.4	37	49	0.725
	Present	252	52.2	99	159		8	8.6	4	4	
LN metastasis (8th edition)	Absent	231	47.8	120	111	0.006	86	91.4	37	49	0.930
	1 ~ 3 LNs	180	37.3	65	115		4	4.3	2	2	
	≥ 4 LNs	72	14.9	28	44		4	4.3	2	2	
LNR	<0.118	323	66.9	154	169	0.039	88	93.6	37	51	0.398
	≥ 0.118	160	33.1	56	104		6	6.4	4	2	
Chemotherapy	No	253	52.4	113	140	0.854	32	34.0	18	14	0.084
	Yes	230	47.6	100	130		62	66.0	23	39	
Radiotherapy	No	342	70.8	148	194	0.615	66	70.2	31	35	0.368
	Yes	141	29.2	65	76		28	29.8	10	18	
TNM 8th stage	IA	46	9.5	25	21	0.067	11	11.6	1	10	0.191
	IB	68	14.1	31	37		24	25.5	12	12	
	IIA	58	12.0	31	27		51	54.3	24	27	
	IIB	143	29.6	61	82		4	4.3	2	2	
	III	168	34.8	73	95		4	4.3	2	2	

*LN, lymph node; LNR, lymph node ratio; TNM, tumor-node-metastasis*.

Additionally, 94 patients from the SYSUCC cohort were included. Similar to patients from the SEER cohort, most patients were younger than 60 years old. Cases that had a moderately-differentiated tumor or absence of LN metastasis, made up most of the patients and more than half of patients had tumors that were larger than 4 cm. A total of 62 (66.0%) patients had received chemotherapy while 28 (29.8%) patients received radiotherapy in this study. The two groups of patients had substantially balanced clinical and pathological variables.

### OS Analysis

**Figure 2:**
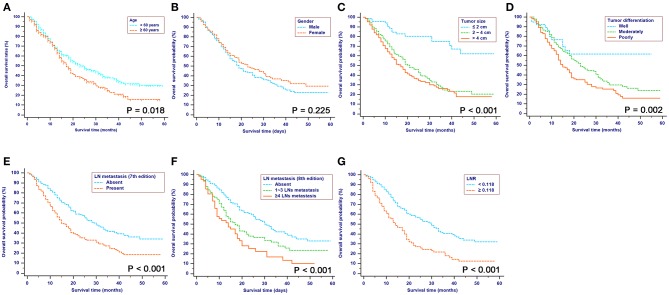
Kaplan-Meier OS curves stratified by patient characteristics: **(A)** Age; **(B)** Gender; **(C)** Tumor size; **(D)** Tumor differentiation; **(E)** LN metastasis (7th edition); **(F)** LN metastasis (8th edition); **(G)** LNR. OS, overall survival; LN, lymph node; LNR, lymph node ratio; TNM, Tumor-Node-Metastasis.

For patients in the SEER cohort, the median OS was 20 months, and the 1-, 2-, and 3-year OS rates were 68.4, 45.7, and 32.8%, respectively. Patients were stratified by characteristics for the OS analyses. The differences in OS rates were all significant, except upon stratification by gender and race (Figure 2 and Table 1). As shown in Table 2, gender, race, and tumor site were not risk factors for OS (*p* > 0.050), while age, tumor size and differentiation, LN metastasis (7th edition), LN metastasis (8th edition), LNR, chemotherapy, and radiotherapy were significantly associated with OS in the univariate analysis. The multivariate analysis, which incorporated these variables, indicated that tumor size, tumor differentiation and LNR sustained their significance in terms of OS after adjusting for covariates. In addition, older patients had a trend for poor OS compared with younger patients, while the multivariate analysis failed to show age as a significant predictor of OS.

**Table 2:** Univariate and multivariate analyses of survival in patients with pancreatic adenocarcinoma of the body and tail.

**Characteristics**		**Overall survival**					
		**Univariate analysis**			**Multivariate analysis**		
		**HR**	**95%CI**	***P***	**HR**	**95%CI**	***P***
Age (years)	<60/≥ 60	1.361	1.049–1.765	0.020	1.222	0.901–1.658	0.197
Gender	Male/Female	0.858	0.667–1.104	0.233			NI
Race	White/Black/Asian	1.078	0.894–1.298	0.432			NI
Tumor site	Body / Tail	1.160	0.900–1.495	0.252			NI
Tumor size (cm)	≤ 2/2 ~ 4/> 4	1.648	1.366–1.988	<0.001	1.539	1.225–1.934	<0.001
Tumor differentiation	Well/Moderately/Poorly	1.492	1.190–1.870	0.001	1.283	1.005–1.637	0.045
LN metastasis (7th edition)	Absent/Present	1.760	1.359–2.278	<0.001	1.093	0.625–1.911	0.756
LN metastasis (8th edition)	Absent/1 ~ 3/≥ 4 LN metastasis	1.564	1.308–1.871	<0.001	0.936	0.625–1.403	0.749
LNR	<0.119/≥ 0.119	2.111	1.604–2.779	<0.001	1.771	1.113–2.818	0.016
Chemotherapy	No/Yes	2.015	1.511–2.889	<0.001	1.585	1.021–2.774	0.001
Radiotherapy	No/Yes	1.178	0.88–2.114	0.184			NI

*HR, hazard ratio; CI, confidence interval; NI, not included*.

### Construction of the Nomogram

**Figure 3:**
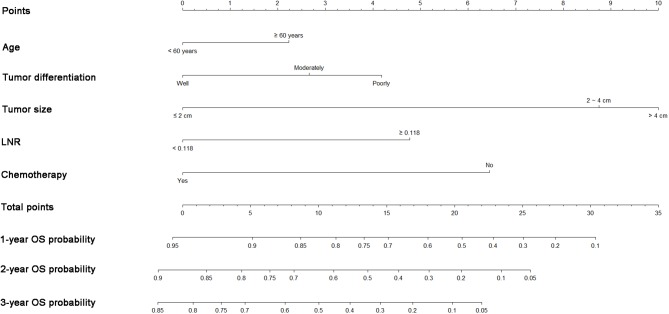
Nomograms predicting 1-, 2-, and 3-year OS of patients with pancreatic adenocarcinoma of the body and tail after surgery. OS, overall survival; LNR, lymph node ratio.

**Figure 4:**
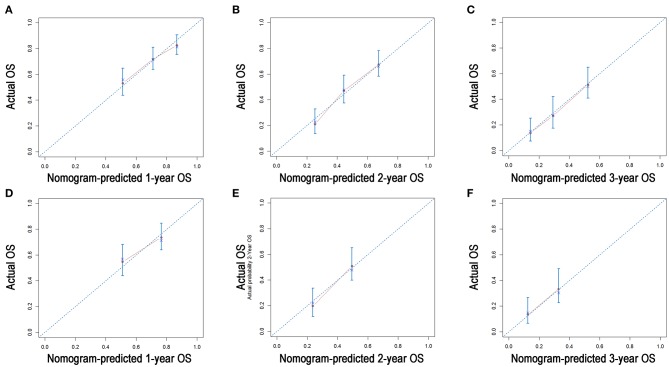
Calibration plots of the nomogram for 1-, 2-, and 3-year OS prediction in the training cohort **(A–C)** and validation cohort **(D–F)**. X-axis represents the nomogram-predicted probability of survival; Y-axis represents the actual OS probability. A perfectly accurate nomogram prediction model would result in a plot that the observed and predicted probabilities for given groups fall along the 45-degree line. Dots with bars represent nomogram-predicted probabilities along with 95% confidence interval.

A nomogram was built using the abovementioned variables *via* the Cox proportional hazards model. As shown in Figure 3, the nomogram predicted the OS probabilities of 1–3 years for patients with PDAC of the body and tail after surgery. For patients from the SEER cohort, the bias-corrected concordance indexes (C-indexes) of the nomogram were higher than those of the 7th edition of the TNM staging system [0.775 (95% CI, 0.731–0.819) vs. 0.617 (95% CI, 0.575–0.659), *P* = 0.006] and the 8th edition of the TNM staging system [0.775 (95% CI, 0.731–0.819) vs. 0.632 (95% CI, 0.588–0.676), *P* = 0.033]. Elevated C-indexes of the established nomogram were also observed in patients from the SYSUCC cohort, compared with the TNM staging system (Table 3). Excellent agreement between the predictive and the actual observed 1-, 2-, and 3-year OS survival was shown by calibration plots (Figure 4). The discriminatory power of the newly developed nomogram was superior to that of the TNM staging system in this study.

**Table 3:** Comparison of the C-index and AUC values between nomograms and TNM stages.

**Patients**		**Overall survival**				
		**C-index**	**AUC**			***P***
			**1-year**	**2-year**	**3-year**	
SEER cohort	Nomogram	0.775 (0.731–0.819)	0.661	0.733	0.721	
	7th TNM stage	0.617 (0.575–0.659)	0.639	0.691	0.667	0.006
	8th TNM stage	0.632 (0.588–0.676)	0.651	0.702	0.690	0.033
SYSUCC cohort	Nomogram	0.747 (0.685–0.809)	0.636	0.682	0.644	
	7th TNM stage	0.602 (0.553–0.651)	0.620	0.646	0.589	0.008
	8th TNM stage	0.607 (0.551–0.663)	0.620	0.652	0.572	0.018

*C-index, concordance index; AUC, area under receiver operating characteristic curves*.

### Comparison of AUC Values of the Nomogram and TNM Staging System

**Figure 5:**
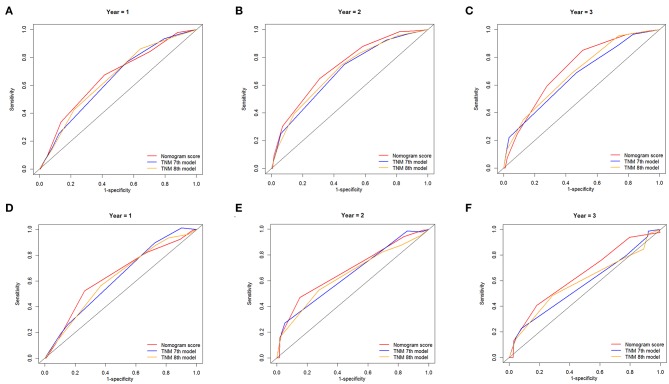
Comparison of the ROC curves of the nomogram and the TNM stage systems for 1-, 2-, and 3-year OS prediction in the training cohort **(A–C)** and validation cohort **(D–F)**.

The ROC curves were used to compare the precision of the 1-, 2-, and 3-year OS predictions. For the training cohort from the SEER database, the AUC values of the nomogram that predicted 1-, 2-, and 3-year OS rates were 0.661, 0.733, and 0.721, respectively, whereas the values of the TNM staging system were 0.639, 0.691, and 0.667 (7th edition) and 0.651, 0.702, and 0.690 (8th edition), respectively. In patients from the external validation cohort, the established nomogram also displayed significantly higher values of AUC than those of the TNM stage system (Table 3). As shown in Figure 5, the nomogram exhibited superior predictive ability to that of the TNM staging system.

## Discussion

The differences in characteristics and prognoses between PDAC of the head and those of the body and tail urged us to pursue a specific staging system for pancreatic body and tail cancer, even though previous studies have compared the predictive power of nomograms in terms of PDAC, (21, 22) which were primarily focused on pancreatic head cancer. In the present study, using a relatively large cohort, a novel nomogram was established to predict OS rates in patients with PDAC of the body and tail in this study. The well-established nomogram seemed to be more significantly predictive than the TNM staging system.

The assessment and prediction of prognosis by the established nomogram differed from those made by current TNM staging systems. Tumor differentiation was proven to be an independent prognostic factor for predicting OS in these patients and was incorporated into the nomogram. Previous studies have confirmed the prognostic significance of tumor differentiation in PDAC, (23, 24) and this was consistent with our results. As a pathological variable which was independent of other variables, including tumor size and LN metastasis, may provide additive prognostic power in survival estimation. According to the nomogram, different levels of tumor differentiation meant different points and different OS rates in patients, even if they had tumors with the same TNM stage. The additive predictive power provided by these variables surely contributed to the superior power of the nomogram for predicting OS, compared with the 7th and 8th editions of the TNM staging system.

Moreover, patients stratified by age had significantly different OS rates in this study. Age alone appeared to affect survival rates after surgical resection in patients with pancreatic cancer (25). Age-related comorbid conditions or complications could possibly contribute to mortality in older patients. Age failed to be classified as an independent prognostic factor, although significant differences in OS were observed among patients stratified by age in this study. It is possible that an even larger cohort will confirm the prognostic significance of age in survival analyses. It was expected that age would have an important role in predicting survival (26). Thus, age was added to the nomogram established in this study and was capable of improving the prognostic efficiency.

Currently, LN involvement remains one of the most important predictors of survival in patients with PDAC (27, 28). An interesting phenomenon was shown in this study: the classic “N” stages of both the 7th and 8th editions of the TNM staging system failed to exhibit independent predictive significance, whereas LNR was proven to be an independent predictive factor in the OS analysis; these results were similar to those of other studies (29, 30). The time-dependent ROC curve analysis was used to determine the optimal cutoff value for LNR. Similar to the results of Pan et al. (31) this method, which was used in many similar reports, (32–34) decided the cutoff value of LNR with more predictive power due to the consideration of the survival time in evaluating the values. As a significant modifier of the effects of LN status, (35) LNR showed the potential for LN metastasis, which was similar with previous studies (36). In this sense, LNR was established as a better predictor of OS than the status of LN metastasis, (37) and a higher LNR value was strongly associated with poor distant metastasis-free survival (38).

In addition to the abovementioned factors, tumor size and chemotherapy were also identified as significant predictors of survival in these patients. Consistent with many previous reports, (21, 39) our results confirmed the significant predictive roles of these two factors in predicting survival in patients with PDAC. Moreover, previous studies had found that status of resection margin of surgery for PDAC of the body and tail had great impact on survival. For a more accurate estimation of survival, all patients included in this study had received radical resection and the impact of resection margin on survival was minimal in this study. Thus, in the nomogram model, each factor from the multivariate Cox proportional hazard regression model was ascribed a weighted point total that implied a survival prognosis. The established nomogram makes it easier for physicians to assess a variety of parameters with more objectivity and precision and to distinguish subgroups with different prognoses among patients with PDAC of the body and tail after radical resection. There are two major applications of the nomogram. First, as a quantitative scoring system, the nomogram can be used to predict survival of patients with PDAC of the body and tail. Second, for patients with high scores calculated by the established nomogram, close follow-up or appropriate treatment may be required. The combination of the main elements from the TNM staging system and other tumor-associated indices, including age, tumor differentiation and LNR, would surely contribute to a better discriminatory power of the nomogram in predicting survival, compared with the TNM staging system. The established nomogram can be used as a practical tool to predict clinical outcomes, and it has potential use in decision-making regarding subsequent treatment of patients with PDAC of the body and tail after surgery.

There are limitations in the present study that should be addressed. First, this was a retrospective study that relied on the SEER database. Some of the potential predictors of survival, such as perineural invasion, lymphovascular invasion, and portal vein involvement, could not be included in the nomogram. Second, although good fitness was demonstrated for the validation in the present study, we should recognize that bootstrapping is only helpful to reduce the overfit bias of the nomogram. More validations using large and independent cohorts are necessary for the present nomogram.

In this study, we analyzed the prognostic data of PDAC of the body and tail using the SEER database. A nomogram for the estimation of 1-, 2-, and 3-year OS was established based on a large study cohort for the first time. The present nomogram can predict the prognosis of patients with PDAC of the body and tail after surgery with considerable accuracy and can help doctors provide highly tailored patient management in the future.

## Data Availability Statement

The datasets generated for this study are available on request to the corresponding author.

## Ethics Statement

The studies involving human participants were reviewed and approved by Institutional Review Board (IRB) of the Sun Yat-sen University Cancer Center. The patients/participants provided their written informed consent to participate in this study.

## Author Contributions

SL designed the project, reviewed and edited the manuscript, respectively. CH, SS, and YZ performed the study selection, data extraction, statistical analyses and wrote the main manuscripts. CH, SS, YZ, and XL contributed to the classification criteria discussion. All authors reviewed the manuscript.

## Conflict of Interest

The authors declare that the research was conducted in the absence of any commercial or financial relationships that could be construed as a potential conflict of interest.

## Supplementary Material

Supplementary Figure 1 The ROC curve of survival based on the changes of LNR value.
