# Supplementary figures and images for: Score for the Overall Survival Probability of Patients With Pancreatic Adenocarcinoma of the Body and Tail After Surgery: A Novel Nomogram-Based Risk Assessment
Source: Front Oncol. 2020 Apr 28;10:590. doi: 10.3389/fonc.2020.00590 (PMC7212341; doi:10.3389/fonc.2020.00590)

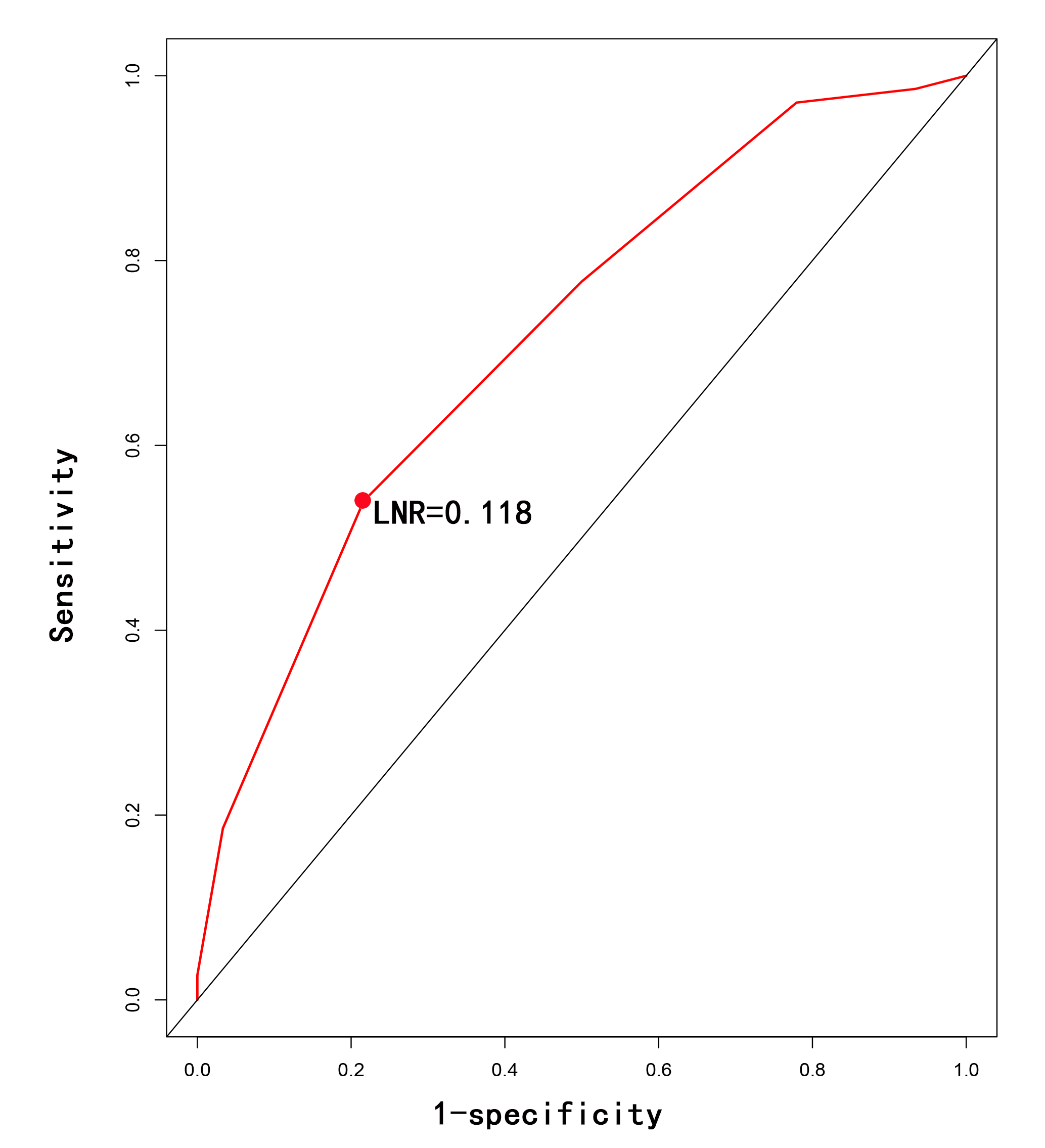

Supplement: Supplementary Figure 1 — The ROC curve of survival based on the changes of LNR value. [file Image_1.TIF]
